# Supplementary material for: Gait alterations in Parkinson’s disease at the stage of hemiparkinsonism—A longitudinal study
Source: PLoS One. 2022 Jul 21;17(7):e0269886. doi: 10.1371/journal.pone.0269886 (PMC9302743; doi:10.1371/journal.pone.0269886)
Supplement: S1 Table — (DOCX) [file pone.0269886.s001.docx]

| Gait parameters | **BASIC GAIT** | | | **MOTOR TASK** | | | **MENTAL TASK** | | | **COMBINED TASK** | | |
| --- | --- | --- | --- | --- | --- | --- | --- | --- | --- | --- | --- | --- |
|  | Symptomatic leg | Asymptomatic leg | p | Symptomatic leg | Asymptomatic leg | p | Symptomatic leg | Asymptomatic leg | p | Symptomatic leg | Asymptomatic leg | p |
| Step time (s) | 0.56±0.044 | 0.55±0.047 | 0.332 | 0.56±0.056 | 0.55±0.057 | 0.288 | 0.62±0.076 | 0.60±0.08 | 0.430 | 0.63±0.08 | 0.62±0.09 | 0.383 |
| Step length (cm) | 61.92±8.87 | 62.65±8.36 | 0.697 | 59.59±8.86 | 60.05±7.89 | 0.805 | 55.94±9.03 | 57.04±8.68 | 0.570 | 53.49±8.63 | 54.13±8.38 | 0.733 |
| Swing time (s) | 0.39±0.025 | 0.39±0.027 | 0.169 | 0.39±0.028 | 0.38±0.026 | 0.123 | 0.42±0.04 | 0.41±0.03 | 0.129 | 0.42±0.04 | 0.41±0.04 | 0.101 |
| Double support time (s) | 0.32±0.064 | 0.32±0.063 | 0.989 | 0.34±0.08 | 0.34±0.08 | 0.982 | 0.40±0.11 | 0.40±0.11 | 0.921 | 0.43±0.13 | 0.43±0.13 | 0.983 |
| CV step time | 4.48±2.29 | 4.54±2.05 | 0.891 | 4.12±1.58 | 3.99±1.39 | 0.680 | 6.50±3.02 | 7.45±6.37 | 0.384 | 6.89±4.38 | 6.57±3.73 | 0.692 |
| CV step length | 4.44±1.57 | 4.26±1.87 | 0.638 | 4.69±2.48 | 4.41±1.99 | 0.571 | 7.38±4.03 | 6.76±3.06 | 0.425 | 7.25±4.49 | 6.57±2.76 | 0.406 |
| CV swing time | 5.12±2.64 | 5.26±3.27 | 0.832 | 5.29±3.41 | 4.75±1.92 | 0.372 | 6.92±3.65 | 6.47±2.66 | 0.514 | 7.57±4.83 | 6.42±3.05 | 0.192 |
| CV double support time | 9.60±4.00 | 9.93±4.19 | 0.714 | 8.85±2.96 | 9.01±3.58 | 0.821 | 13.73±7.18 | 12.63±5.06 | 0.418 | 13.45±7.49 | 12.96±7.09 | 0.759 |
| Velocity (m/s) | 1.14±21.28 | 1.14±21.19 | 0.975 | 1.10±22.48 | 1.10±22.23 | 0.993 | 96.00±23.26 | 95.93±23.48 | 0.989 | 89.72±22.66 | 89.96±22.63 | 0.962 |
| Heal-to-heal base support (cm) | 9.67±2.50 | 9.68±2.52 | 0.984 | 9.39±2.37 | 9.40±2.45 | 0.978 | 10.04±3.06 | 10.07±3.08 | 0.968 | 9.81±2.90 | 9.83±2.91 | 0.975 |

**Supplementary table 1. Gait parameters at baseline of symptomatic leg vs. asymptomatic leg at study entry.**

Values are shown as mean ± SD

Abbreviation: CV – coefficient of variation
