# Supplementary material for: Gait alterations in Parkinson’s disease at the stage of hemiparkinsonism—A longitudinal study
Source: PLoS One. 2022 Jul 21;17(7):e0269886. doi: 10.1371/journal.pone.0269886 (PMC9302743; doi:10.1371/journal.pone.0269886)
Supplement: Attachment — Submitted filename: Markovic et al Response to Reviewers.docx [file pone.0269886.s002.docx]

Federica Provini

Academic Editor

PLOS ONE

**PONE-D-22-04031**

Title: **Gait alterations in Parkinson’s disease at the stage of hemiparkinsonism – a longitudinal study**

Dear Prof. Provini,

Herewith I am enclosing the list of changes against each point that has been raised by the Reviewer, as requested. We hope our manuscript is now improved.

Sincerely,

Vladana Markovic

**Reviewer' comments:**

1) In line 146, it is mentioned that: “Progression of motor and non-motor symptoms, decreased functioning in activities of daily living and higher LED were observed in patients with PD at t1 compared to t0”: how were the clinical assessment of non-motor symptoms and ADL performed? From what is written in the methodology section, I assume this information was extracted from the UDPRS I & II, but could the authors please be more specific in the Methodology?

**Thank you for your suggestion. We changed part of Methodology in the line 88-89, and added clarification in results in line 146-147. We accepted your suggestions and it is now more specific and more precise.**

2) In lines 148-150, it is mentioned that “None progressed to more advanced motor stages or developed motor complications (neither dyskinesia, nor motor fluctuations)”, which means that UDPRS IV should be zero (0) for t0 and t1. However, in table 1, it seems that UDPRS IV is not zero neither in t0 nor in t1, could the authors please elaborate a little more on this?

**UDPRS IV is not zero neither in t0 nor in t1 because several patients experienced painful early morning dystonia. This is now explained in line 152. Thank you for pointing this out.**

3) I would suggest adding the measurement units wherever applicable e.g. in table 2 presenting step time or step length.

**Measurement units for time and length are added in the Table 2 and 3 as well as in the Results section.**

4) I would suggest adding a citation for the GAITRite walkway in order to certify its use as a validated tool for gait assessment.

**Citation for the GAITRite walkway is added (Menz et al, 2014) as additional reference. Reference list is corrected and now we have 27 instead of 26 references.**

5) In line 209, could the authors please be a little more detailed on what does psychiatric worsening mean? Are they referring to the worsening in depressive symptoms?

**Yes, we were referring to depressive symptoms. This is now corrected in the manuscript in the same line.**

6) In the discussion part, the authors mention that despite what has been observed in other studies assessing early PD patients, in this current study no worsening has been shown in gait parameters like velocity or stride length (lines 215-218). Would the authors consider the level of exercise or changes in weight between t0 and t1 as factor that might have contributed to these results?

**We did not considered level of exercise or weight change between t0 and t1 as factor. This is now added in the limitations of the study, line 254.**

7) In line 256, it is mentioned that patients did not complain of gait impairment, even when parkinsonism became bilateral at t1 (H&Y=2): was there any particular question assessing the subjective patient’s impression on gait impairment or an objective measurement that led the authors to this conclusion? Maybe the relevant questions in UDPRS about gait impairment?

**Thank you for your suggestion. In line 256 we made comment on t0 data, suggesting that patients did not complain of gait difficulties at study entry. These data were collected at study entry during history collecting, and findings were shown in results section lines 144-147. Actually, as there were 7,1% of patients that did report gait difficulties, we have corrected this sentence to be accurate. Additionally, we have added a comment in methodology regarding this issue (line 88). We did not imply that patients had not complained of gait difficulties at t1, this was not assessed.**

8) In line 232, it is mentioned that “swing time variability under dual task condition exhibited even greater increase at the initially asymptomatic bodyside”, while in line 257, it is mentioned that “These alterations progress similarly, or even at faster pace, at initially affected bodyside”: could the authors please specify whether the increase was greater in the symptomatic or asymptomatic side during the combined task?

**Thank you very much for bringing this issue up, we have actually made an error in line 257 (now 265 after corrections), it should have been written “at initially unaffected bodyside”. We apologize for this mistake.**

9) In line 181, it is mentioned that “spatial-temporal parameters recorded at initially asymptomatic bodyside suggested shortening of the swing time and its CV during all test conditions at t1 compared to t0”: did the authors mean that CV was increased and not shortened?

**Thank you for this comment, we have corrected this line according to your suggestion.**

**Editor’ comments:**

Please upload your figure files to the Preflight Analysis and Conversion Engine (PACE) digital diagnostic tool. PACE helps ensure that figures meet PLOS requirements.

**Figure 1 is uploaded to the Preflight Analysis and Conversion Engine (PACE) digital diagnostic tool. Now, the figure meets PLOS requirements.**

If there are no restrictions, please upload the minimal anonymized data set necessary to replicate your study findings as either Supporting Information files or to a stable, public repository and provide us with the relevant URLs, DOIs, or accession numbers.

**We added data set in the repository. Please find the data in the following link:** [**https://www.kaggle.com/datasets/vladanamarkovic/gait-alterations-in-hemiparkinsonism**](https://www.kaggle.com/datasets/vladanamarkovic/gait-alterations-in-hemiparkinsonism)

We note that you have included the phrase “data not shown” in your manuscript. Unfortunately, this does not meet our data sharing requirements. PLOS does not permit references to inaccessible data. We require that authors provide all relevant data within the paper, Supporting Information files, or in an acceptable, public repository. Please add a citation to support this phrase or upload the data that corresponds with these findings to a stable repository (such as Figshare or Dryad) and provide and URLs, DOIs, or accession numbers that may be used to access these data.

**Thank you for your suggestion. Instead of “data not shown” we added the data in the supplementary table, now it is Supplementary table 1, and it is uploaded. Table title is: Gait parameters at baseline of symptomatic leg vs. asymptomatic leg at study entry.**
